# Frequency multiplexing enables parallel multi-sample EPR

**DOI:** 10.1038/s41598-024-62564-4

**Published:** 2024-05-23

**Authors:** Chun Him Lee, Jan G. Korvink, Mazin Jouda

**Affiliations:** Karlsruhe Institute of Technology, Institute of Microstructure Technology, 76344 Eggenstein-Leopoldshafen, Germany

**Keywords:** Parallel EPR, Parallel ODNP, High-throughput, Electrical and electronic engineering, Analytical chemistry

## Abstract

Electron paramagnetic resonance (EPR) spectroscopy stands out as a powerful analytical technique with extensive applications in the fields of biology, chemistry, physics, and material sciences. It proves invaluable for investigating the molecular structure and reaction mechanisms of substances containing unpaired electrons, such as metal complexes, organic and inorganic radicals, and intermediate states in chemical reactions. However, despite their remarkable capabilities, EPR systems face significant limitations in terms of sample throughput, as current commercial systems only target the analysis of one sample at a time. Here we introduce a novel scheme for conducting ultra-high frequency continuous-wave EPR (CW EPR) targeting the EPR spectroscopy of multiple microliter volume samples in parallel. Our proof-of-principle prototype involves two decoupled detection cells equipped with high qualty factor $Q=104$ solenoidal coils tuned to 488 and 589 MHz, ensuring a significant frequency gap for effective radio frequency (RF) decoupling between the channels. To further enhance electromagnetic decoupling, an orthogonal alignment of the coils was adopted. The paper further presents an innovative radiofrequency circuit concept that utilizes a single physical RF channel to simultaneously conduct parallel EPR on up to eight cells. Parallel EPR experiments on two BDPA samples, each with a sample volume of 18.3 μL, registered signal-to-noise ratios of 255 and 252 for the two EPR measurement cells, with no observable coupling. The showcased prototype, built using cost-effective commercially available fabrication technology, is readily scalable and represents an initial step with promising potential for advancing sample screening with high-throughput parallel EPR.

## Introduction

Electron paramagnetic resonance (EPR) is a general technique with which to characterize materials containing unpaired electrons. For example, the detection of reactive oxygen species (ROS) found in plants and mammals with EPR, which can reveal types and quantities of ROS, provides crucial findings for the interaction between ROSs and pathogens^1–3^. The application of ROS detection stretches to tumor^4^, cancer^5^ and diabetes studies^6^. Another stream for EPR spectroscopy in the junction of spin trapping and spin labeling creates a powerful tool to reveal the structure and the conformational dynamics of proteins.^7,8^ In more recent applications, researchers have been actively developing a technique to locate the coronavirus fusion peptide (FP) using EPR spectroscopy, to identify and quantify paramagnetic biosamples^9,10^, a crucial tool for understanding coronaviruses infecting humans.

In general, EPR has become an important analytical tool in multiple research fields, and the only tool for unambiguously detecting these free radicals^11,12^. Specifically, in chemistry^13–15^, biology^11,16,17^, material science^18–21^, and medical research^1–3,9,10^, critical spectral data, particularly about metal complexes (i.e., Cu^2+^, Fe^3+^ and Mn^2+^ complexes) and organic radicals, are provided by studying unpaired electromagnetically (EM) excited electrons of the samples of interest under action of an oscillating EM wave $B_1$ perpendicular to the static magnetic field $B_0$. Radiofrequency (RF) or microwave resonators play a crucial role in EPR spectrometry, serving as essential components that actively stimulate and detect the electron spins in the sample. The electrical properties of these resonators, including their quality factor (also referred to as the Q-factor), return loss, and sensitivity (represented as $B_1/(i\cdot \sqrt{R})$, where *i* is the current, and *R* represents the losses), directly impact the performance of EPR experiments by influencing detection aspects such as bandwidth and signal-to-noise ratio (SNR).

A common EPR frequency segment that has been widely used is the X-band (8–12 GHz), leading to commercial implementation of various detector systems designed to operate within this range. These spectrometers are often tailored to optimize sensitivity^22^, sample size^23^, bandwidth^24^, and filling factor^25^. Notably, certain types of detectors have demonstrated exceptional capabilities in concentrating the $B_1$ field needed for excitation and detection. Examples include the cavity resonator, known for its high sensitivity^26^, and the microstrip line resonator, designed for a high filling factor^25^.

Despite its tremendous capabilities and the significant benefits it offers across various applications, EPR is still limited by its low throughput capability, restricting analysis to one sample at a time. This limitation becomes especially significant when factoring in the time required for pre-measurement preparations, which encompass tasks such as sample preparation, sample loading, and system calibration. For applications such as diagnosis or study of a disease, and developing suitable drugs for it, the constrained throughput capacity of EPR poses a substantial challenge in comprehending for example the peptides associated with the action of a virus and, consequently, hinders the expedited development of appropriate drugs and vaccines.

In this paper, we introduce a novel approach to enable parallel EPR spectroscopy experiments by developing a two-detector-cell system allowing the simultaneous measurement of two samples and serving as a first step towards high-throughput EPR spectroscopy. The measurement cells comprise two orthogonally aligned solenoids tuned to UHF frequencies of 488 φand 589 MHz, and placed at the center of an EPR magnet such that their $B_1$ fields are mutually perpendicular to $B_0$. The paper additionally introduces a smart readout scheme that benefits from the multi-carrier homodyning capabilities of a digital lock-in amplifier to eliminate the RF coupling between the two EPR cells while utilizing a single readout physical channel. The paper then reports the parallel EPR measurement results and finally highlights the potential to scale up this concept without additional hardware costs.

## Materials and methods

### Parallel detector design

**Figure 1:**
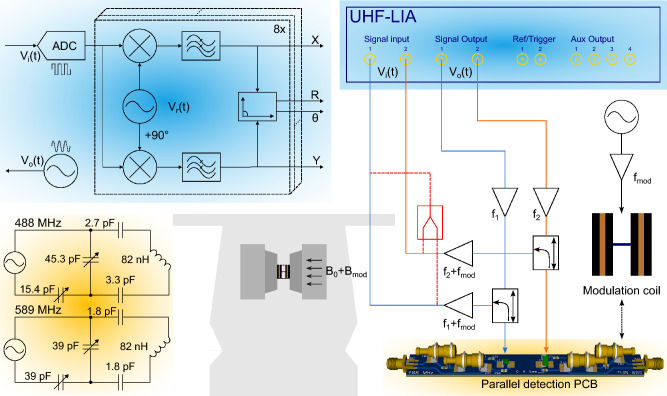
Schematic diagram of the parallel EPR setup. The lock-in amplifier, highlighted in blue, utilizes digital modulation and demodulation where up to eight software demodulators can be freely and independently assigned to the digitized RF input signal. Two excitation signals are coupled to the EPR detectors through directional couplers which then direct the reflected waves carrying the EPR signals to the RF inputs of the lock-in amplifier. The red section of the connection diagram refers to an alternative hardware-efficient scheme, allowing parallel signals to be read out using a single RF input channel. The parallel detection PCB and the corresponding tuning and matching networks are highlighted in yellow, and on the left indicate the orthogonality of the two detector coils.

**Figure 2:**
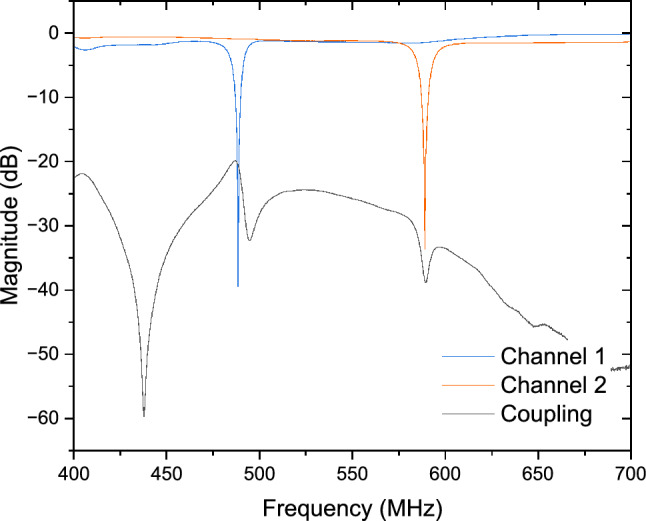
Unloaded reflection and transmission coefficients of the parallel detector. The Q factor of the circuits for the two channels was measured to be 104.7 and 104.2, respectively.

Figure 1, highlighted in yellow, explains the prototype design of the two-port parallel EPR detector set. It comprises two orthogonally placed high Q air core inductors (1515SQ-82N, Coilcraft, Inc.), a matching network with high Q chip capacitors (KEMET Electronics GmbH), and high Q non-magnetic sapphire trimmer capacitors (V9000, Knowles Voltronics). The resonant matching network functions as a passive amplifier, reducing the noise impact of the RF receiver. The two detectors operate at close but different frequencies, with a frequency separation of 100 MHz approximately, ensuring that the static magnetic field $B_0$ interacts with the two samples during the field sweep while eliminating the RF coupling between the coils. The RF coils allow for a solid or liquid sample, prepared with a cylindrical capillary up to the diameter of the air core (1.8 mm). Moreover, the solenoids are placed 20 mm apart, which, in addition to permitting orthogonal placement relative to each other and to $B_0$, guarantees sufficient electromagnetic decoupling of − 20 dB as shown in Fig. 2, thereby enabling a reliable parallel operation. A single-port tuning scheme was adopted for each RF coil, wherein the incident wave to the port serves to excite the electron spins while the reflected wave carries the EPR signal as a modulating signal. Impedance matching, as demonstrated in Fig. 1, follows a straightforward T-matching network^27^, consisting of a combination of fixed-value and variable capacitors to maintain the Q-factor and ensure efficient use of the trimmers’ tuning range.

The circuit was modeled and optimized in Advanced Design System (ADS), where an S-parameter simulation over the range 400–600 MHz was used to analyze the port reflection (S11) in the mentioned one-port system. Inductor models in the series with different values can be found, which offers more design flexibility. All measured S-parameters models were available online from the manufacturer (Coilcraft, Inc.) for a realistic simulation. An inductor with the highest Q was selected for optimized sensitivity.

### Helmholtz coil design

Taking advantage of the short longitudinal relaxation time $T_1$, field modulation, where the $B_0$ is varied over a relatively high-frequency range of 20–100 kHz during the sweep, is commonly employed in EPR to (1) enhance the detection sensitivity by allowing lock-in phase-sensitive acquisition, (2) improve the spectral resolution through applying different modulation frequencies to resolve the closely separated peaks^28^, and (3) amplify the detected EPR signal, thereby boosting the detection limit. As the available magnet is not equipped with a built-in modulation coil, we had to design a custom field modulation coil. The Helmholtz-based coil in Fig. 1, consists of 150 windings per half, made of an isolated copper wire of diameter 250 μm, resulting in an inductance of 1.4 mH. The diameter of the coil is 4.5 cm with an accessible space of 1.5 cm.

### Lock-in modulation and heterodyning

Employing heterodying within the digital lock-in detection process enables efficient signal processing resulting in minimal circuit complexity for parallel measurements. The reference signals for excitation, $f_1$ and $f_2$, are generated using the RF output ports of the lock-in amplifier. Then, a Helmholtz coil that modulates $B_0$ with frequency $f_{\text {mod}}$, results in the EPR signal appearing in the reflected wave as a modulating signal carried by a $f_1+f_{\text {mod}}$ or $f_2+f_{\text {mod}}$ carrier. This signal is then input into the lock-in amplifier where its internal demodulation frequencies are set as the heterodyned frequencies. Figure 1 highlights in blue the eight internal demodulators of the lock-in amplifier that can independently demodulate the EPR signals. The application of heterodyning through Helmholtz coil modulation significantly boosts the EPR signal’s strength, preparing it for further processing. Once amplified, the signals are introduced to the inputs of the lock-in amplifier. Finally, the digitization process operates at a rate at least 3 times greater than the Nyquist criterion within the lock-in amplifier, with a constant sampling frequency of 1.8 Gsps, guaranteeing a lossless demodulation phase. Sampling and converting the mixed signal into a digital format before demodulation enables the isolation and extraction of the specific signal amongst potential noise or interference sources. By oversampling the signal, the digital representation captures the details of the original signal greater than the Nyquist rate, allowing post acquisition signal filtering and reconstruction, therefore, providing an accurate demodulation where the signal is mixed with the heterodyned frequency. This approach reduces circuit complexity while enhancing the signal-to-noise ratio (SNR), allowing for more efficient and accurate parallel EPR measurements.

### Manufacturing and test setup

The PCB prototype was fabricated in-house using a standard PCB fabrication process based on chemical etching of a 1.5 mm mm FR4 single-sided substrate. The ultimate version of the PCB was then manufactured via a commercial PCB service. A solid-state sample (1,3-bisdiphenylene-2-phenylallyl, BPDA) and a liquid-state sample ((2,2,6,6-Tetramethylpiperidin-1-yl)oxyl, TEMPO) were chosen for measurement, for benchmarking the EPR detectors and to characterize their stability. In addition, we could expect a distinguishable single-peak spectrum from BDPA, and a three-peak spectrum from TEMPO, to facilitate the assessment of potential cross-coupling between the signals. The first sample was purchased readily in powder form. For the second sample, TEMPO was first dissolved in ethanol. Both samples were transferred to a glass or Kapton-made capillary tube, and sealed with UV glue after loading the sample. Consequently, the sealed sample tube could be loaded into the coil without leakage.

The experiment for parallel EPR detection is schematically illustrated in Fig. 1. The setup consisted of a 600 MHz UHF lock-in amplifier (UHFLI, Zurich Instruments), a programmable DC power supply (72-2540, TENMA), a wideband amplifier (ZX60-P103LN+, Mini-Circuits), a low-noise MMIC amplifier (LNA, ZX60-P103LN+, Mini-Circuits), a directional coupler (ZX30-12-4-S+, Mini-Circuits) for each channel, a modulation Helmholtz coil, a high-frequency high-voltage amplifier (Trek 2100HF, Advanced Energy) for the Helmholtz coil, and an EPR electromagnet (ELEXSYS-II E500 CW-EPR, Bruker BioSpin). The 10^′′^ magnet has a gap of 72 mm and can be swept from 0 T to 1.45 T. Additionally, it features a field homogeneity of 10 mG over a length of 22 mm along the sample axis. The lock-in amplifier generated continuous sinusoidal excitation waves at its two RF output channels, with frequencies $f_1$ and $f_2$, which underwent amplification before being coupled to the resonators. Moreover, an additional function generator was synchronized with the lock-in amplifier to provide a low frequency $f_\text {mod}={30}$ kHz signal for the modulation Helmholtz coil, with an AC current driven by the high-voltage amplifier. Note that all electronic modules were shielded to minimize electromagnetic interference.

**Figure 3:**
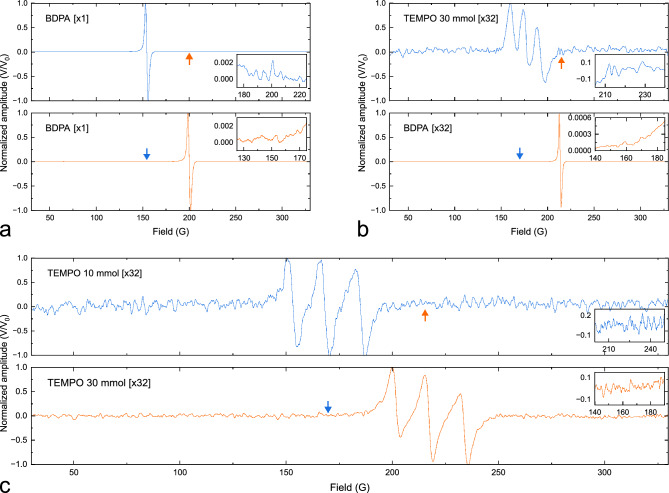
Parallel EPR measurements for different experimental scenarios. (**a**) BDPA and BDPA, measured with an input signal voltage of 80 mV, modulation amplitude of 200 mV, and modulation frequency of 30 kHz, in a single shot. (**b**) TEMPO (30 mmol) and BDPA, measured with input signal voltage of 80 mV for 30 mmol TEMPO and 40 mV for BDPA, modulation amplitude of 100 mV, and modulation frequency of 30 kHz, averaged with 32 scans. (**c**) TEMPO 10 mmol and TEMPO 30 mmol, measured with input signal voltage of 80 mV, modulation amplitude of 200 mV, and modulation frequency of 30 kHz, averaged with 32 scans. Phase correction of the EPR spectrum was performed in Matlab. Coupling between two channels is denoted with an arrow and an insert zoomed-in view of the spectral response, revealing no observable coupling.

## Results

A sequence of parallel EPR experiments was conducted to validate and characterize the performance of the introduced setup. In each trial, two samples were loaded into glass capillaries with an inner diameter of 1.8 mm. Subsequently, the loaded samples were inserted into the EPR detectors, which were, in turn, mounted within the custom modulation coil positioned inside the magnet. Utilizing the frequency sweep function of the UHFLI, we were able to readjust the tuning and matching of the detectors following disturbances caused by the loading effects of the samples. To ensure reliable operation of the parallel setup in terms of sensitivity, and complete elimination of crosstalk across various experimental scenarios, a solid-state BDPA sample and a series of liquid-state TEMPO samples with different concentrations were employed. These scenarios encompassed using two BDPA samples that featured strong EPR signals, as depicted in Fig. 3a, to confirm the absence of coupling even at high excitation signal levels, where the measured SNR of the two samples registered 255 and 252 respectively. Conversely, Fig. 3b depicts a scenario where sample 1 is a BDPA with a strong EPR signal, while sample 2 is a 30 mmol TEMPO sample with a signal 1000 times weaker. This necessitated boosting the internal amplifier of channel 2 of the UHFLI to enhance sensitivity. Remarkably, even at this heightened sensitivity level for sample 2, no observable coupling from the strong signal of sample 1 could be measured. Figure 3c illustrates a scenario where both samples exhibit weak EPR signals, in which case the internal sensitivity of the lock-in amplifier’s RF input channels is set to their maximum. Similar to the other scenarios, this verifies the complete elimination of coupling between the channels. During the measurements, the main field $B_0$ was swept from 30 G to 330 G, the modulation frequency was set to 30 kHz, and the scan time was set to 10 s with a lowpass filter bandwidth of 8 Hz for the digital lock-in amplifier. The lock-in lowpass filters utilized were of third order offering a – 60 dB/decade signal attenuation outside the 8 Hz passband. The excitation and field modulation amplitudes varied depending on the experiment. However, a maximum excitation amplitude of 80 mV and a maximum field modulation amplitude of 200 mV, corresponding to an average modulation field of 4 G, was used. The sampling rate after the lowpass filter stage was set to 8 times over the Nyquist sampling rate, and 1–32 scans were performed in various experiments to perform signal averaging. Moreover, a TTL trigger signal from the UHFLI was used to synchronize the data acquisition with the $B_0$ field sweep that was controlled via the Bruker “Xepr” software. This synchronization facilitated the direct correlation of the field sweep and the acquisition time axis, and consequently made signal averaging possible. In all experiments, the UHFLI’s built-in software “LabOne” was used to perform the measurements, while Matlab was employed for subsequent post-processing and phase correction.

**Figure 4:**
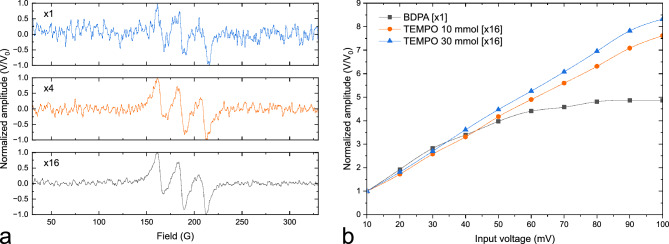
Experiments that necessitate signal averaging and multi-parameter-sweep are examples of experiments that would particularly benefit from parallelization. (**a**) Normalized result of automated signal averaging $(\times 1, \times 4, \times 16)$ on a sample sample of 30 mmol TEMPO. (**b**) Automated concurrent power sweep experiment on multiple samples. The excitation amplitude was swept from 10 to 100 mV and the results were normalized to 10 mV. Each TEMPO data point in the figure was obtained from averaging 16 scans.

In EPR experiments on samples of particularly low concentrations of analyte, signal averaging becomes crucial to enhance the signal-to-noise ratio and thus boost the measurement sensitivity. This enhancement is at the expense of prolonged measurement times. Figure 4a illustrates an example where parallel EPR is particularly invaluable to perform simultaneous signal averaging on multiple samples, enabling a significant reduction in overall scan time. As an alternative, one can accelerate a single sample averaging experiment by loading the same sample with identical volume into all the detectors. The individual spectra can then be aligned offline and averaged. Therefore, an *n*-scan-averaging task, having a time complexity of $t_n$, can be divided into the number of channels $m_\textit{par}$ and the number of concurrent scans $n_\textit{seq}$, where $n = n_\textit{seq}*m_\textit{par}$. The SNR would be increased by the factor $\sqrt{n} = \sqrt{n_\textit{seq}*m_\textit{par}}$. The time required would be significantly reduced to the number of available channels, where $t_n = t_1*n/m_\textit{par} = t_1*n_\textit{seq}$, $t_1$ being the time of one concurrent scan. Figure 4b, on the other hand, depicts an example of an even more time-demanding scenario where parallel EPR can also be extremely beneficial. In this figure, a two-dimensional sweep with 16 averages was conducted to find the optimum excitation power corresponding to the maximum SNR for different samples. These samples pairs comprised BDPA—30 mmol TEMPO, and 10 mmol TEMPO—30 mmol TEMPO. The peak-to-peak output voltage was normalized to an initial value of 10 mV. Regression can be observed for BDPA starting from 60 mV, while linear relations of input voltage against peak-to-peak output voltage were imposed for TEMPO from 10 mV to 100 mV.

### Hardware-efficient readout scheme

**Figure 5:**
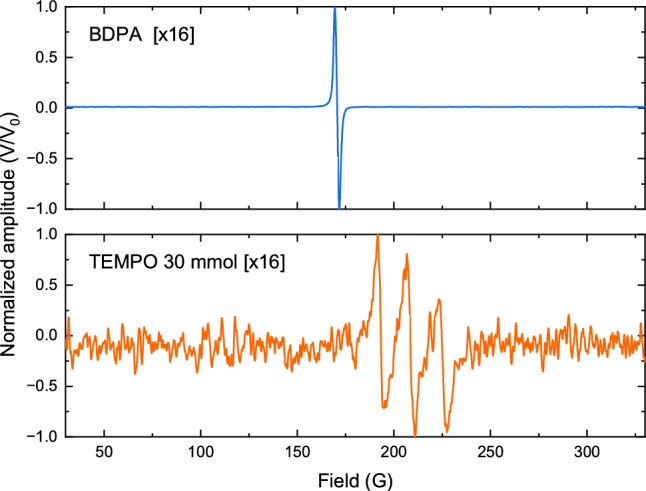
Result of a parallel EPR experiment in which combined signals from two samples were read out by a single RF input channel, and subsequently digitally separated.

Since the two EPR detectors operate at different frequencies, we can combine their signals by adding a combiner (ZFSC-2-4-S+, Mini-Circuits) after the low-noise amplifiers, as highlighted in red in Fig. 1. The output of the combiner, containing a frequency multiplexed version of the two EPR signals, is then acquired by a single RF channel of the UHFLI. Utilizing eight independent software digital demodulators, this hardware-efficient readout scheme can be straightforwardly applied to acquire a frequency multiplexed EPR signal from eight parallel detectors. Figure 5 depicts the results of the simultaneous measurements of two EPR samples utilizing a single receive channel. Thanks to the frequency separation between the detectors, and the powerful lock-in capability of the UHFLI, the EPR spectra of the two samples were faithfully separated, impressively with no observable crosstalk.

## Discussion

This paper introduces a novel approach enabling simultaneous EPR measurement of multiple samples, toward highly accelerated and high-throughput parallel EPR spectroscopy. A proof-of-concept of the method was demonstrated using two miniaturized EPR detectors operating at the UHF frequency. Conducting EPR at this low frequency indeed offers a unique opportunity to increase the detector size (due to the larger wavelength), consequently allowing a larger sample volume and thereby a higher SNR, as nicely outlined by Biller et al.^29^ and Rinard et al.^30^. However, the choice of miniaturized detectors in our case was mainly due to the limited space inside the magnet with high B0 homogeneity, and the potential of microcoils to boost the sensitivity to mass-limited samples as illustrated by Narkowicz et al.^31^ and Boero et al.^32^

Taking advantage of the frequency offset between detectors, and employing digital lock-in detection, the method enables concurrent acquisition of EPR signals from multiple samples with no measurable crosstalk, even when signals were multiplexed and acquired using a single RF channel. Although we adopted an orthogonal alignment of the coils to ensure sufficient decoupling, the remarkable suppression of crosstalk in the presented results was predominantly achieved by the powerful lock-in capability of the UHFLI benefiting from the large frequency difference (100 MHz) between the two channels. Furthermore, the use of digital lock-in detection with software-based demodulators provides a unique scalability advantage, allowing increasing the number of parallel detectors without requiring additional hardware. The method described is generalizable and thus holds great potential for many application fields of EPR spectroscopy. More specifically, the envisioned applications of the presented concept involve detecting reactions and processes that produce radicals in extremely low concentrations. Typically, such detection requires extensive averaging where many scans are needed to enhance the signal-to-noise ratio (SNR). Therefore, conducting such experiments simultaneously on multiple samples would significantly reduce the experiment time. Moreover, experiments that require multi-dimensional scans or sweeping experimental parameters (e.g. sample temperature, illumination conditions, irradiation dose, etc.) can profit largely from parallelization. Additionally, comparative analysis of concentration measurements against a control sample can benefit from simultaneous measurements where it is ensured that the measurement of different samples depends merely on sample properties like concentration, molecular structure, and relaxation time, and all other experimental nonidealities, such as environmental fluctuations as well as user errors in sample loading and resonator tuning, can be neutralized. Therefore, a rapid and reliable comparative parallel environment allows, for instance, the investigation of spin-trapping efficiency, or radical concentrations of samples at slightly different fields, providing access to a potentially more comprehensive understanding of reaction kinetics, radical formation, or reaction pathways^33–35^. Although the proposed parallel EPR concept was demonstrated and validated at the UHF frequency range, it can, nevertheless, be readily applied to X-band and even higher frequency ranges, considering the advancements in analog-to-digital converter (ADC) technology, and the existence of high-speed digitizers such as the AD9084 from Analog Devices® with 20 Gsps, or the 8-bit ADC from Fujitsu® with 56 Gsps.

## Data Availability

The data and material that support the findings of this study are available from the corresponding author upon request. Alternatively, the dataset can be accessed from this DOI: 10.35097/KefAuwQWRQPRPiur.
